# Efficacy of Debridement, Antibiotics, and Implant Retention for Periprosthetic Joint Infections Following Hip and Knee Arthroplasty: A Retrospective Cohort Study

**DOI:** 10.7759/cureus.84591

**Published:** 2025-05-21

**Authors:** Arfaz Shaik, Arjun S Chakrapani, Umar Hayat, Ameen Ahmed, Dheeraj Panchaksharam Selvarajan, Nafsheen Shaik

**Affiliations:** 1 Trauma and Orthopaedics, Croydon University Hospital, London, GBR; 2 Trauma and Orthopaedics, Wrightington Wigan and Leigh NHS Foundation Trust, Wigan, GBR; 3 Trauma and Orthopaedics, Torbay and South Devon NHS Foundation Trust, Devon, GBR; 4 Orthopaedics, Portsmouth Hospitals University NHS Trust, Portsmouth, GBR

**Keywords:** antibiotics therapy, debridement and implant retention, implant retention, orthopedic implant-related infection, periprosthetic joint infections

## Abstract

Background: This study aimed to determine how well debridement, antibiotics, and implant retention (DAIR) work for treating infections after total hip and knee replacements. Specifically, it compared the success of this treatment in resolving infection, improving function, and preventing complications when the infection occurred early (within three months) versus late (three months to 12 months) after the joint replacement surgery.

Objective: The objective of the study is to evaluate DAIR’s efficacy in resolving periprosthetic joint infections (PJIs) post-THAs (total hip arthroplasties) and TKAs (total knee arthroplasties), focusing on infection resolution, functional recovery, and complications.

Methods: This retrospective cohort study reviewed 37 patients (25 TKA, 12 THA) treated with DAIR for PJIs at the UK tertiary centre (2019-2023). PJIs were diagnosed using the 2018 International Consensus Meeting (ICM) criteria.Outcomes (infection status, range of motion (ROM), pain, complications) were assessed via electronic health records, with a mean follow-up of 12.8 months for TKA and 10.4 months for THA. The statistical analysis tool used was IBM SPSS Statistics for Windows, Version 26 (Released 2019; IBM Corp., Armonk, New York, United States).

Results: DAIR successfully treated infection in 73.9% of infected total knee replacements and 58.3% of infected total hip replacements (p=0.576). Early infections (within three months) responded better to DAIR (88% for TKA, 91.7% for THA) compared to late infections (0% success, p<0.01). Complications arose in nine TKA and eight THA patients (p=0.157), including three deaths, which were more frequent in late infections (p=0.04). Following DAIR, there were significant reductions in average C-reactive protein levels for both TKA (from 134.5 to 16.9 mg/L, p<0.01) and THA (from 147.1 to 41.6 mg/L, p = 0.03).

Conclusion: DAIR achieved infection resolution in 73.9% of TKA and 58.3% of THA cases, particularly when performed within three months of infection onset, while late infections (>three months) had 0% success, emphasising the need for timely intervention and patient selection.

## Introduction

Periprosthetic joint infections (PJIs) complicate approximately 0.5%-2% of total hip arthroplasties (THAs) and total knee arthroplasties (TKAs), with reported incidences of 0.3%-1.9% for THAs and 0.8%-1.9% for TKAs [1]. These infections pose significant challenges, impairing joint function, increasing patient morbidity, and substantially elevating healthcare costs due to prolonged treatments and potential revision surgeries [2]. Debridement, antibiotics, and implant retention (DAIR) has emerged as a less invasive alternative to revision surgery for managing early PJIs, aiming to preserve the prosthesis while eradicating infection [3]. However, its success rate varies widely (20%-89.5%), influenced by factors such as infection timing, surgical technique, and microbiological profile [4].

Recent literature highlights DAIR’s potential efficacy in early infections [5]. Kunutsor et al. [6] reported favourable outcomes in systematic reviews, while Grammatopoulos et al. [7] demonstrated success rates exceeding 70% in early cases, contrasting with poorer results in chronic infections. This variability underscores the need for studies to refine DAIR’s role in clinical practice. A study conducted by Quinn et al. at a UK tertiary centre over a three-year period (2019-2023) evaluated DAIR’s efficacy in resolving PJIs post-THA and TKA, focusing on infection resolution, functional recovery, and complications [5]. We hypothesise that DAIR is effective for early PJIs (<three months post-arthroplasty) but less so for late infections (>three months), providing evidence to guide treatment decisions in orthopaedic surgery.

## Materials and methods

This retrospective cohort study assessed the efficacy of DAIR for PJIs following TKA and THA, employing descriptive and inferential statistical analyses to evaluate outcomes across a diverse patient cohort. As this study was a retrospective analysis of pre-existing data, and there was no direct contact or interaction with patients, formal ethical clearance was deemed unnecessary. All the medical records were deidentified for the purpose of the study, with all the necessary permissions.

Setting

Data were collected from electronic health records at a UK tertiary orthopaedic centre between January 2019 and December 2023. PJI diagnosis adhered to the 2018 International Consensus Meeting (ICM) criteria [8] incorporating clinical signs (e.g., sinus tract), laboratory markers (e.g., CRP, synovial fluid analysis), and microbiological confirmation.

Participants

This study included 37 adult patients (25 with infected total knee replacements and 12 with infected total hip replacements) who had PJIs confirmed by established criteria and were treated with DAIR. These 37 cases were selected from a larger group of 89 PJI cases identified (45 TKA, 44 THA) during the study. To be included, patients needed complete initial information and at least six months of follow-up. Patients with incomplete data or those who received different treatments, such as one- or two-stage revision surgery, were not included. Patient enrollment was consecutive, mirroring typical clinical practice, with an average follow-up of 12.8 months for TKA patients and 10.4 months for THA patients (ranging from 6 to 24 months). Due to the uncommon nature of PJIs, the number of patients in this study was limited, making a formal sample size calculation impractical. However, the researchers conducted sensitivity analyses to check the reliability of the main findings.

Intervention

DAIR procedures followed a standardised protocol: open arthrotomy, thorough debridement of infected tissue, irrigation with ≥9 L saline, and, where indicated, exchange of modular components (e.g., polyethylene liners in TKA, femoral heads in THA). Local antibiotics (Stimulan, Biocomposites Ltd., UK) were applied in 56% (14/25) of TKA and 58.3% (7/12) of THA cases, using calcium sulfate beads with vancomycin or gentamicin based on culture sensitivity. Intravenous antibiotics were administered for 6-12 weeks post-DAIR, tailored to microbiology, followed by oral suppression where needed. Surgeons with >10 years of experience performed all procedures, ensuring consistency.

Variables

The primary outcome was infection status at follow-up, defined as the absence of clinical signs (e.g., sinus tract), CRP <10 mg/L, and sterile culture results, consistent with ICM 2018 guidelines. Secondary outcomes included TKA ROM (range of motion: degrees, physical exam), pain (categorised as nil, mild, moderate, severe), and complications (e.g., reoperation, death). Exposures included infection timing (<3 vs. >3 months), microbiological profile, and surgical variables (e.g., component exchange). Confounders, age, sex, BMI, ASA grades [9], and time to DAIR, were recorded to contextualise outcomes. The unit of analysis was the patient (one joint per case; no bilateral cases occurred).

Data sources and measurement

Data were extracted from electronic health records, including operative notes, laboratory results, and outpatient follow-ups. Infection status was triangulated using clinical assessment, CRP, white blood cell count (WBC), and microbiology. Physiotherapists measured ROM using goniometry; pain was patient-reported. Missing data (1 microbiology) underwent sensitivity analysis, assuming an observed distribution, with negligible impact on conclusions.

Bias

Selection bias (DAIR in 55.6% TKA, 27.3% THA) was minimised by applying ICM criteria uniformly. Information bias was reduced through dual verification of records by two researchers.

Statistical analysis

Descriptive statistics (SDs) included means for normally distributed data (e.g., age, CRP), medians (ranges) for skewed data (e.g., time to infection), and frequencies (percentages). Inferential tests-Chi-square, Fisher’s exact (for small samples), and paired t-tests-assessed differences (IBM SPSS Statistics for Windows, Version 26 (Released 2019; IBM Corp., Armonk, New York, United States); p<0.05), with 95% confidence interval (CIs) reported. Normality was assessed visually using histograms and confirmed where necessary for parametric tests; independence was assumed given single-joint analysis. Significance was set at 5%. Subgroup analyses explored timing, microbiology, and surgical factors, with odds ratios (ORs) calculated for key associations (e.g., S. aureus and failure).

Ethics

The study was registered with the centre’s Clinical Audit Department. Per NHS Health Research Authority guidelines, no ethics approval was required as it involved retrospective analysis of anonymised standard-care data, and strict confidentiality was maintained throughout.

## Results

Study population

Of 89 PJI patients, 37 underwent DAIR (25 TKA, 12 THA). TKA patients had a mean age of 68.8 years (SD 12.0) (17 male patients/eight female patients), a mean BMI of 34.2 kg/m^2^ (SD 7.8), a median ASA score of 2 (range 1-4), a mean time to infection of 127.9 weeks (median 28, range 1-800), and a mean time to DAIR of 8.1 days (median 4, range 1-65). THA patients had a mean age of 72.0 years (SD 8.9) (two male patients/10 female patients), a mean BMI of 33.7 kg/m^2^ (SD 7.6), median ASA 2 (range 1-3), a mean time to infection of 7.5 weeks (median 4, range 3-36), and a mean time to DAIR of 22.6 days (median 7, range 2-180). Baseline differences (e.g., shorter time to infection in THA) reflect distinct clinical presentations (Table 1).

**Table 1:** Demographic and clinical characteristics of patients undergoing debridement, antibiotics, and implant retention, by joint type. THA: Total Hip Arthroplasty; TKA: Total Knee Arthroplasty; BMI: Body Mass Index; ASA: American Society of Anesthesiologists; DAIR: Debridement, Antibiotics, and Implant Retention

Parameters	Age (Years)		Sex (Male/Female)		BMI		ASA Score		Time of Infection (Week)			DAIR (Days)		
Details of Parameters	Mean	SD	M	F	Mean BMI (kg/m^2^)	SD	Median	Range	Mean	Median	Range	Mean Time	Median	Range
TKA (n=25)	68.8	12.0	17	8	34.2	7.8	2	1-4	127.9	28	1 - 800	8.1	4	1 - 65
THA (n=12)	72.0	8.9	2	10	33.7	7.6	2	1 -3	7.5	4	3 - 36	22.6	7	2 - 180

Functional outcomes

In TKA (n=21 with ROM data), postoperative ROM varied: 47.6% (10/21) achieved 0-110°, 14.3% (3/21) 0-90°, 9.5% (2/21) 30-60°, and others ranged from 0 to 125° to arthrodesis. Pain outcomes (n=22) measured by the visual analogue score [10] included 59.1% (13/22) with no pain, 22.7% (5/22) mild, 9.1% (2/22) moderate, and 9.1% (2/22) severe (p=0.15, Fisher’s exact test vs. THA). In THA (n=12), pain outcomes were 58.3% (7/12) none, 25% (3/12) mild, and 16.7% (2/12) severe (p=1.00). The analysis compared postoperative mobility between total hip replacement (THR) and total knee replacement (TKR) patients using a chi-square test with Yates' continuity correction. While a higher percentage of TKR patients (60%) were independently mobile compared to THR patients (50%), and more THR patients required walking aids like sticks or scooters, the differences in mobility distribution were not statistically significant (p = 0.208). This suggests that there is no meaningful difference in postoperative mobility outcomes between THR and TKR groups based on this sample. Mobility in TKA showed 60% (15/25) as independent, 12% (3/25) using a stick, 16% (4/25) using crutches, and 4% (1/25) using a scooter (p<0.001, chi-square vs. assisted mobility), reflecting significant functional recovery in early cases (Table 2). Pre-DAIR pain and ROM were markedly worse (e.g., mean TKA ROM 45°, SD 20°), improving post-DAIR (p<0.001, paired t-test), underscoring DAIR’s rehabilitative potential when successful.

**Table 2:** Postoperative mobility distribution between THR and TKR. A chi-square test with Yates' continuity correction was applied for more than 2X2 contingency tables to estimate the p-value. THR: Total Hip Replacement; TKR: Total Knee Replacement

	THR (n=12)	TKR (n=25)	p-value
Independent	6(50.0%)	15(60.0%)	0.208
Walking with stick	3(25.0%)	3(12.0%)	
Walking with crutches	0	4(16.0%)	
Using a scooter	1(8.30%)	0	
Difficult to walk	1(8.30%)	0	

Microbiological findings

TKA cultures revealed *Staphylococcus aureus* in 28% of cases (7/25), polymicrobial infections in 24% of cases (6/25), and no growth in 16% of cases (4/25). THA cultures showed S. aureus in 33.3% of cases (4/12), polymicrobial infections in 16.7% of cases (2/12), and no growth in 16.7% of cases (2/12) (p=1.00, Fisher’s exact test). Polymicrobial infections had lower odds of resolution (OR=0.39, 95% CI 0.09-1.62, p=0.27) compared to monomicrobial infections, though not statistically significant due to sample size constraints. *S. aureus* was strongly associated with DAIR failure (OR=3.2, 95% CI 1.1-9.5, p=0.03), highlighting its virulence in PJIs. Sensitivity analysis excluding missing microbiology data (n=1) confirmed these trends, with polymicrobial rates in TKA (42.2% of positive cultures) notably higher than THA (18.2%), suggesting distinct infection profiles.

Surgical details

Component exchange occurred in 84% (21/25) of TKA and 50% (6/12) of THA cases. In TKA, exchange involved polyethylene liners; in THA, it included femoral heads or acetabular liners based on intraoperative findings. Patients with exchange showed a trend toward higher resolution (85.7% [6/7] vs. 20% [1/5] without, p=0.072, Fisher’s exact test, 95% CI -0.05 to 0.85), particularly in THA (Table 3). Local antibiotics (Stimulan, Biocomposites Ltd., UK) were used in 56% of TKA cases (14/25) and 58.3% of THA cases (7/12), with no significant impact on outcomes (p=1.00, Fisher’s exact test). Cemented (8/12), hybrid (3/12), and uncemented (1/12) THA implants showed no difference in resolution (p=0.444-0.491, pairwise Fisher’s exact tests), though small numbers limit inference.

**Table 3:** Impact of component exchange on infection resolution in total hip arthroplasty patients; Fisher’s exact test was used to estimate the p-value.

Component Exchange	Infection Resolved	Persistent Infection	p-value
No	1(14.3%)	4(80.0%)	0.072
Yes	6(85.7%)	1(20.0%)	

Infection outcomes

Component exchange was strongly associated with infection resolution, occurring in 85.7% of cases where it happened versus only 14.3% when it did not (Table 3). Despite this clear trend, the difference was not statistically significant (p=0.072), likely attributable to the small sample size (N=12). The majority of PJIs in this study involved TKA for both early (88%) and late (12%) infections (Table 4), and THA for early (91.7%) and late (8.3%) infections; this distribution was not statistically significant (p=0.82). The primary indication for TKA was septic knee (64%), while for THA, it was serous discharge with a sinus tract (27.3%). Infection resolution rates were 73.9% for TKA and 58.3% for THA (p=0.45). Notably, early PJIs (<3 months) showed high success rates in both TKA (88%) and THA (91.7%), whereas late PJIs (>3 months) had 0% success (p<0.01). Significant decreases in mean C-reactive protein (CRP) and white blood cell (WBC) count were observed post-treatment in both TKA and THA groups (all p<0.03). Furthermore, a higher American Society of Anesthesiologists (ASA) grade was significantly associated with a lower likelihood of infection resolution (p=0.0059).

Complications

Complications affected nine TKA patients (36%): amputation (2), arthrodesis (1), deep vein thrombosis (DVT) (1), stiff knee (2), long-term antibiotics (2), unstable knee (1), and death (2). In THA, eight patients (66.7%) experienced complications: Girdlestone procedure (3), foot drop (2), chronic back pain (2), and death (1) (p=1.00, Fisher’s exact test, 95% CI -0.28 to 0.28). Deaths (3 total) were linked to late PJIs or complex cases without exchange (p=0.04, 95% CI 0.02-0.36), with two TKA patients succumbing to sepsis and one THA patient to multiorgan failure. Wound healing showed 80% (20/25) TKA healed vs. 58.3% (7/12) THA (p=1.00), with chronic sinuses in unhealed cases. Complication rates underscore the stakes of DAIR failure, particularly in late or high-risk patients. A summary of key outcomes is presented in Table 4.

**Table 4:** Summary of key outcomes following debridement, antibiotics, and implant retention, by joint type. THA: Total Hip Arthroplasty; TKA: Total Knee Arthroplasty; PJIs: Periprosthetic Joint Infections

Parameter	TKA (n=25)	THA (n=12)
Early PJI (%)	88%	91.7%
Infection resolved (%)	73.9%	58.3%
Component exchange (%)	84%	50%
Complications (n)	9	8
Deaths (n)	2	1

Subgroup analyses

Early PJI success was robust across ASA grades 1-2 (90%, 18/20) but dropped to 50% (3/6) in grades 3-4 (p=0.0059). Age negatively impacted resolution (p=0.02, t-test), with patients >70 years showing 55% success (11/20) vs. 88% (14/16) in younger patients. BMI (>30 kg/m^2^) had no significant effect (p=0.99), though trends suggested higher complications in obese patients (50% vs. 33%, p=0.16). Time to DAIR (<7 days vs. >7 days) showed no clear impact (p=0.36), though early intervention trended toward better outcomes (80% vs. 65%).

## Discussion

Our findings confirm the previously reported efficacy of DAIR for early PJIs, with a 73.9% success rate in TKA and 58.3% in THA. These findings align closely with Grammatopoulos et al. [7] (72.2%) and Barros et al. [11] (89.5% early success). Early cases (<3 months) achieved 88% (TKA) and 91.7% (THA) resolution, while late PJIs (>3 months) failed entirely (0%, p<0.01), mirroring Shield et al. [12] (45% chronic failure). This dichotomy likely stems from biofilm maturation in late infections, which resists debridement and systemic antibiotics, as Osmon et al. [13] and Sen et al. [14] note in their guidelines. The TKA-THA gap may reflect anatomical differences-knee infections often involve superficial soft tissues amenable to debridement, whereas hip infections penetrate deeper, per Yoshihara et al. [15]. This suggests that joint-specific DAIR protocols could optimise outcomes.

Component exchange significantly reduced complications (p=0.04), with 85.7% success vs. 20% without, corroborating Grammatopoulos et al. [16] (84% with exchange). Exchange likely removes contaminated modular components, disrupting biofilms and enhancing antibiotic penetration, a critical insight for surgical decision-making. Microbiologically, TKA’s 42.2% polymicrobial rate (vs. 18.2% THA) aligns with Hu et al. [17] reflecting knee exposure to skin flora or hematogenous spread. S. aureus’s link to failure (OR=3.2, p=0.03) underscores its tenacity, per Scheper et al. [18] necessitating aggressive management (e.g., rifampicin combinations) in such cases. These findings advocate for advanced diagnostics like next-generation sequencing to guide therapy, especially in TKA.

Functionally, DAIR restored significant outcomes-60% of TKA patients regained independence, and pain relief was notable (59.1% TKA, 58.3% THA pain-free). Pre- to post-DAIR improvements in ROM (p<0.001) and inflammatory markers (CRP/WBC, p<0.01) highlight its efficacy in early successes, offering patients a less invasive path to recovery. However, complications (36% TKA, 66.7% THA) and deaths (8.1%, 3/37) in late or complex cases reveal DAIR’s limits. Older age (>70, p=0.02) and higher ASA grades (p=0.0059) predicted poorer outcomes, while BMI trends (p=0.16) suggest obesity complicates recovery, per Longo et al. [3].

Clinical implications

DAIR is a cornerstone for early, stable PJIs, particularly TKA, with THA success enhanced by exchange and rapid intervention (<3 months). Surgeons should prioritise exchange in THA and consider revision for late PJIs, avoiding delays that increase morbidity. The high polymicrobial burden in TKA demands preoperative synovial cultures and broad-spectrum antibiotics, potentially reducing failures. For high-risk patients (e.g., elderly, ASA >2), multidisciplinary assessment could triage DAIR vs. revision, optimising resource use and patient outcomes. Implementing these strategies could lower revision rates, healthcare costs, and patient burden, aligning with value-based care models.

Strengths and limitations

Strengths include ICM diagnostic rigor, comprehensive outcomes (infection, function, complications), and real-world consecutive enrolment. Single-centre consistency, with experienced surgeons, minimises technical variability. Limitations include the small cohort (n=37), reducing power for rare events (e.g., deaths), and missing data (1 microbiology), though sensitivity analyses mitigated impact. Retrospective design limits causality, and single-centre data may not reflect diverse settings. Follow-up (mean 12.8 months TKA, 10.4 months THA) captures short-term outcomes, but late recurrences remain unassessed.

Future directions

Multi-centre studies with larger cohorts could confirm THA efficacy and explore microbiology (e.g., polymicrobial resistance patterns). Randomised trials comparing DAIR with vs. without exchange, or vs. revision in intermediate cases (3-6 months), could define optimal thresholds. Long-term follow-up (>5 years) would assess durability, particularly for functional gains. Innovations like higher-dose local antibiotics or biofilm-disrupting agents could boost success, addressing current failures. Integrating machine learning with diagnostic and outcome data might predict DAIR success, personalising treatment.

## Conclusions

This study confirms DAIR's efficacy for early PJIs, especially in TKA, aligning with prior research. Timely intervention (<three months) is crucial for success, contrasting with complete failure in late infections, likely due to biofilm maturation. Component exchange significantly boosts THA outcomes, suggesting its importance in disrupting biofilms. The TKA-THA success gap may reflect anatomical differences. Higher polymicrobial rates in TKA and link of* S. aureus *to failure necessitate tailored antimicrobial approaches. Functionally, DAIR improves pain and mobility in early successes, but complications rise in delayed or complex cases, with older age and higher ASA grades predicting poorer outcomes. These findings underscore the need for early intervention, component exchange in THA, and advanced diagnostics to optimise patient selection and outcomes. Future research should focus on multi-centre validation and innovative strategies to enhance DAIR's success, particularly in challenging scenarios.

## References

[1] Dobson PF, Reed MR (2020). Prevention of infection in primary THA and TKA. EFORT Open Rev.

[2] Ayoade F, Li D, Mabrouk A, Todd JR (2025). Periprosthetic joint infection. StatPearls [Internet].

[3] Longo UG, De Salvatore S, Bandini B, Lalli A, Barillà B, Budhiparama NC, Lustig S (2024). Debridement, antibiotics, and implant retention (DAIR) for the early prosthetic joint infection of total knee and hip arthroplasties: a systematic review. J ISAKOS.

[4] Qasim SN, Swann A, Ashford R (2017). The DAIR (debridement, antibiotics and implant retention) procedure for infected total knee replacement - a literature review. SICOT J.

[5] Quinn J, van Duren BH, Berber R, Higgins M, Matar HE, Manktelow AR, Bloch BV (2025). Efficacy of adjunctive antiseptic lavage solution in managing acute hip/knee prosthetic joint infection: a comparative study in a tertiary revision center. Arthroplast Today.

[6] Kunutsor SK, Beswick AD, Whitehouse MR, Wylde V, Blom AW (2018). Debridement, antibiotics and implant retention for periprosthetic joint infections: a systematic review and meta-analysis of treatment outcomes. J Infect.

[7] Grammatopoulos G, Bolduc ME, Atkins BL (2017). Functional outcome of debridement, antibiotics and implant retention in periprosthetic joint infection involving the hip: a case-control study. Bone Joint J.

[8] Sigmund IK, Luger M, Windhager R, McNally MA (2022). Diagnosing periprosthetic joint infections : a comparison of infection definitions: EBJIS 2021, ICM 2018, and IDSA 2013. Bone Joint Res.

[9] Hendrix JM, Garmon EH (2025). American Society of Anesthesiologists Physical Status Classification System. StatPearls [Internet].

[10] Haefeli M, Elfering A (2006). Pain assessment. Eur Spine J.

[11] Barros LH, Barbosa TA, Esteves J, Abreu M, Soares D, Sousa R (2019). Early Debridement, antibiotics and implant retention (DAIR) in patients with suspected acute infection after hip or knee arthroplasty - safe, effective and without negative functional impact. J Bone Jt Infect.

[12] Shield WP 3rd, Greenwell PH, Chapman DM, Dalury DF (2019). Ignore the patella in revision total knee surgery: a minimum 5-year follow-up with patella component retention. J Arthroplasty.

[13] Osmon DR, Berbari EF, Berendt AR (2013). Diagnosis and management of prosthetic joint infection: clinical practice guidelines by the Infectious Diseases Society of America. Clin Infect Dis.

[14] Sen CK, Roy S, Mathew-Steiner SS, Gordillo GM (2021). Biofilm management in wound care. Plast Reconstr Surg.

[15] Yoshihara H, Hasegawa K, Okamoto M, Hatsushikano S, Watanabe K (2018). Relationship between sagittal radiographic parameters and disability in patients with spinal disease using 3D standing analysis. Orthop Traumatol Surg Res.

[16] Grammatopoulos G, Kendrick B, McNally M (2017). Outcome following debridement, antibiotics, and implant retention in hip periprosthetic joint infection: an 18-year experience. J Arthroplasty.

[17] Hu L, Fu J, Zhou Y, Chai W, Zhang G, Hao L, Chen J (2023). Microbiological profiles and antibiotic resistance of periprosthetic joint infection after hip replacement in patients with fracture or non-fracture: a comparative study. J Back Musculoskelet Rehabil.

[18] Scheper H, Gerritsen LM, Pijls BG, Van Asten SA, Visser LG, De Boer MG (2021). Outcome of debridement, antibiotics, and implant retention for staphylococcal hip and knee prosthetic joint infections, focused on rifampicin use: a systematic review and meta-analysis. Open Forum Infect Dis.

